# Ear your heart: transcutaneous auricular vagus nerve stimulation on heart rate variability in healthy young participants

**DOI:** 10.7717/peerj.14447

**Published:** 2022-11-21

**Authors:** Giuseppe Forte, Francesca Favieri, Erik Leemhuis, Maria Luisa De Martino, Anna Maria Giannini, Luigi De Gennaro, Maria Casagrande, Mariella Pazzaglia

**Affiliations:** 1 Body and Action Lab, IRCCS Fondazione Santa Lucia, Rome, Italy; 2 Department of Psychology, University of Roma “La Sapienza”, Rome, Italy; 3 Dipartimento di Psicologia Clinica, Dinamica e Salute, University of Roma “La Sapienza”, Rome, Italy

**Keywords:** Heart rate variability, Vagus nerve, Transcutaneous auricular vagus nerve stimulation, taVNS, tvns, Cymba conchae, Cardiac activity

## Abstract

**Background:**

Transcutaneous auricular vagus nerve stimulation (taVNS) stimulating the auricular branch of the vagus nerve along a well-defined neuroanatomical pathway, has promising therapeutic efficacy. Potentially, taVNS can modulate autonomic responses. Specifically, taVNS can induce more consistent parasympathetic activation and may lead to increased heart rate variability (HRV). However, the effects of taVNS on HRV remain inconclusive. Here, we investigated changes in HRV due to brief alteration periods of parasympathetic-vagal cardiac activity produced by taVNS on the cymba as opposed to control administration *via* the helix.

**Materials and Methods:**

We compared the effect of 10 min of active stimulation (*i.e*., cymba conchae) to sham stimulation (*i.e*., helix) on peripheral cardiovascular response, in 28 healthy young adults. HRV was estimated in the time domain and frequency domain during the overall stimulation.

**Results:**

Although active-taVNS and sham-taVNS stimulation did not differ in subjective intensity ratings, the active stimulation of the cymba led to vagally mediated HRV increases in both the time and frequency domains. Differences were significant between active-taVNS and both sham-taVNS and resting conditions in the absence of stimulation for various HRV parameters, but not for the low-frequency index of HRV, where no differences were found between active-taVNS and sham-taVNS conditions.

**Conclusion:**

This work supports the hypothesis that taVNS reliably induces a rapid increase in HRV parameters when auricular stimulation is used to recruit fibers in the cymba compared to stimulation at another site. The results suggest that HRV can be used as a physiological indicator of autonomic tone in taVNS for research and potential therapeutic applications, in line with the established effects of invasive VNS. Knowledge of the physiological effect of taVNS short sessions in modulating cardiovagal processing is essential for enhancing its clinical use.

## Introduction

Among non-invasive forms of brain stimulation (NIBS), transcutaneous auricular vagus nerve stimulation (taVNS) is a rapidly developing neuromodulatory technique that offers unique opportunities for modulating vagal afferents *via* the widespread system of connections of the nucleus tractus solitarius (NTS) (Farmer et al., 2021). Simple, safe, and inexpensive, taVNS has quickly gained popularity (Butt et al., 2020) as a promising new therapeutic approach for treating neurological and psychiatric disorders (De Martino et al., 2021; Stegeman et al., 2021; Wu et al., 2018). According to empirical data, taVNS stimulates a variety of autonomic, emotional, and cognitive processes involved in the afferent vagal pathway that terminates in the NTS and forebrain (Berlau & McGaugh, 2006; Berthoud & Neuhuber, 2000; Chamberlain et al., 2006; Colzato et al., 2013; Van Leusden, Sellaro & Colzato, 2015).

Despite the emerging consensus, the reliability of commonly taVNS protocols has been questioned, and the mechanisms of action are only partially defined at the moment, mainly considering controversial reports or null taVNS-related changes (Borges et al., 2021; Keute et al., 2021; Warren et al., 2019).

Therefore, taVNS protocols often do not apply a crossover design or have adopted numerous variations (Badran et al., 2018a; Burger et al., 2019) for the stimulation target of the auricular branch of the vagus nerve (cymba conchae and the tragus targets (Butt et al., 2020; Colzato et al., 2018a)). Other parameters, such as current intensity, pulse width, and stimulation duration, also vary.

Similarly, the physiological processes underlying the effects of taVNS remain largely unknown (Burger et al., 2020a; Wolf et al., 2021). Excitation of afferent fibers of the peripheral vagus nerve triggers brainstem activity that promotes cardiac activity (Badran et al., 2018b; Rush et al., 2000). Increased efferent vagal activity excites neurons projecting to the sinoatrial node of the heart to release acetylcholine, which decreases the heart rate and increases vagal-mediated heart rate variability (vmHRV) (Burger et al., 2020a). Rapid beat-to-beat variations, such as root mean square of successive differences (RMSSD) and high-frequency power (HF, 0.15–0.40 Hz), reflect vmHRV (Laborde, Mosley & Thayer, 2017) as an indirect measure of (efferent) vagal function. However, recent studies of vmHRV as a biomarker of taVNS have yielded controversial results, often reporting multiple null findings, as suggested in a Bayesian meta-analysis by Wolf et al. (2021), which has cast doubt on the reliability of the marker of autonomic tone.

Several studies have reported an increase in vmHRV (Antonino et al., 2017; Clancy et al., 2014; Lamb et al., 2017; Tran et al., 2019), whereas others have found no increase (Burger et al., 2020a; Burger et al., 2019; Teckentrup et al., 2020). Kaniusas et al. (2019) questioned the effects of taVNS on vmHRV (Kaniusas et al., 2019). Burger et al. (2020b) and Butt et al. (2020) did not find solid evidence in their reviews and meta-analyses and concluded that there is no evidence to support the hypothesis that vmHRV is a reliable biomarker for online taVNS. Even narrative reviews on the modulation of vmHRV are inconclusive (Wolf et al., 2021). Possible explanations for the heterogeneity of the effects may be attributed to the excitation of afferent fibers *via* taVNS that innervate the heart indirectly *via* brainstem nuclei, rather than directly (De Couck et al., 2017; Kaniusas et al., 2019; Safi, Ellrich & Neuhuber, 2016; Vuckovic, Tosato & Struijk, 2008). The reviews also emphasized significant methodological differences between studies (*e.g*., experimental design and implementation, optimal stimulation sites and parameters, and reported HRV parameters). Considering that the stimulation has shown beneficial effects in the reduction of symptoms in psychopathology (*e.g*., depression (Fang et al., 2016; Koenig et al., 2021)) and in other medical conditions (*e.g*., heart failure, pain, epilepsy) (Aihua et al., 2014; Napadow et al., 2012; Stavrakis et al., 2020), it is critical to understand its potential for research and therapeutic applications as well as to evaluate its limitations. From a safety perspective, besides alternating on-off stimulations, vulnerable patients could be subjected to short sessions of taVNS (De Martino et al., 2021). Consequently, the aim of this study was to evaluate how taVNS stimulation applied to the cymba conchae and helix affects vmHRV parameters after a brief session of 10 min. Clinical applicability depends on knowledge of the physiological role of taNVS in modulating cardiovagal processing. If taVNS increases HRV, our work can be used as a comprehensive reference to advance future treatment of diseases with low HRV.

## Materials and Methods

### Participants

Sample size was determined using G*Power 3 software (Faul et al., 2007).

The computation parameters included setting α to 0.05, desired power (1 − β) to 0.95, and a moderate effect size (d = 0.25) expected for the effects of taVNS on HRV based on a previously published study (Wolf et al., 2021). Accordingly, a sample size of at least 25 was estimated. However, considering the potential for cardiac rhythm alteration, a sample of 30 participants was considered adequate.

Participants were eligible if they were between 18 and 30 years of age, right-handed, free of extensive ear piercings, and not taking medications that might affect the autonomic nervous system.

According to the guidelines (Farmer et al., 2021), participants also had no contraindications to taVNS: (a) pregnancy; (b) active implants (*e.g*., pacemakers, cochlear implants) or brain shunts; (c) previous neurological or psychiatric diagnoses; (d) history of addiction or substance abuse; (e) trauma and/or brain surgery; (f) cardiac disease; (g) acute or chronic use of medications and/or drugs; (h) susceptibility to headaches and seizures.

Of the 30 participants included in the study, two individuals who reported altered HRV values with high noise levels were excluded from data analysis of the study. A total of 28 participants (mean age: 23.15 years ± SD = 3.16, 23 female) in condition of normal weight (BMI < 25.0) completed the study. All participants had to be naive to the purposes and experimental procedure. No participant had previously received any type of vagal stimulation. Data were collected from September 2021 to January 2022.

The protocol was approved by the Ethics Committee of the University of Rome “La Sapienza” (Protocol number: 0001541), and all participants signed the informed consent. Recruitment of participants took place on a voluntary basis (dissemination of advice) at “Sapienza” University of Rome. Prior to each experimental session, to voluntaries were given some general information of the study and the instructions to follow in order to comply with the International Guidelines for the Assessment of HRV (Malik, 1996): abstaining from nicotine and caffeine consumption in the two 2 h before the assessment and from alcohol consumption and intense physical activity in the 12 h before the experiment.

### Auricular transcutaneous vagus nerve stimulation

The taVNS® L device developed by tVNS Technologies GmbH (Erlangen, Germany) was used. The approved device (CE certification) consists of a programmable stimulation unit connected to two titanium electrodes located in an earphone-like structure. Stimulation was applied to the left ear to avoid stimulation of fibers to the heart (Kreuzer et al., 2012; Sperling et al., 2010) and parameters are pre-set, with a biphasic impulse frequency delivered at a rate of 25 Hz, width of 200–300 μs and an on-off cycle of 30 s (Borges, Laborde & Raab, 2019; De Couck et al., 2017).

For the active taVNS condition, the electrode was placed at the cymba conchae, which has been shown to have the highest density of projections from the auricular branch of the vagus nerve (Badran et al., 2018b; Peuker & Filler, 2002; Safi, Ellrich & Neuhuber, 2016) and to cause greater activation of the vagal pathway (Yakunina, Kim & Nam, 2017a). For the active sham condition, electrodes were placed at the helix without overlapping the other innervation areas, which are not expected to elicit vagal activation (Ellrich, 2019; Peuker & Filler, 2002). Stimulation loci were cleaned with alcohol cotton swabs to reduce skin resistance, and the electrode head was placed according to the characteristics of the participants’ ears.

### Cardiac vagal activity

The Firstbeat Bodyguard two heart rate monitoring system (Firstbeat Analytics, Jyväskylä, Finland) was adopted to assess cardiac vagal activity. The signal was acquired by two Ag/AgCl electrodes (Ambu BlueSensor L, Ballerup, Denmark), one applied under the right collarbone and the other under the left rib cage. Both the time and frequency domains were analyzed. The time domain included the standard deviation of the RR intervals (SDNN) and the square root of the mean of the square of successive differences between adjacent R-R intervals (rMSSD). In the frequency domain, the low-frequency range (LF; 0.04–0.15 Hz), reflecting a mix of sympathetic and vagal influences, and the high frequencies (HF; 0.15–0.40 Hz), an index of the parasympathetic cardiac tone (Laborde, Mosley & Thayer, 2017), were considered. The ratio of power in these frequency bands, LF/HF, was calculated. However, it is important to consider that the ratio may be influenced by different aspects (*e.g*., body position), complicating the use of the LF /HF as a reliable index of parasympathetic-sympathetic balance (Billman, 2013; von Rosenberg et al., 2017). HRV signals were analyzed using Kubios software (Tarvainen et al., 2014) (ver. 3.4.3., Kubios Oy, Kupio, Finland) and adopting a custom correction according to previous studies and HRV guidelines (Forte, Favieri & Casagrande, 2019; Forte et al., 2021; Laborde, Mosley & Thayer, 2017). This procedure allows the exclusion of possible outlier measurements. For each participant, HRV was evaluated at rest (baseline) and during active/sham stimulation conditions.

### Self-reported adverse reaction

Potential side effects of the applied stimulation were assessed at the time of stimulation and at the conclusion of the stimulation periods by asking participants to report their taVNS-related sensations. Each sensation was rated on a numerical rating scale, from 0 (not at all) to 100 (the highest unpleasant sensation/very high feeling of tension). A baseline requirement for the procedure was that the stimulation was perceptible but not disturbing or painful. At the end of the experiment, participants were asked to inform the investigators about possible side effects to avoid interference from unpleasant/painful sensations and to ensure an appropriate level of comfort. In addition, screening for any adverse effects was also ensured between the two sessions and at the end of the procedure for any problems that may arise, but no side effects were reported.

### Procedure

To remove inter-subject variability from the comparison between groups frequently encountered in HRV analysis, we adopted a sham-controlled, single-blinded, randomized crossover within-subject design (Quintana & Heathers, 2014) that reduced the effect of covariates, for instance, age and gender related effects on HRV (Bretherton et al., 2019; Clancy et al., 2014; Deuchars et al., 2018; Koenig et al., 2021). In order to ensure greater comparability of the cardiac signal, the two experimental sessions were scheduled at the same hour and day of the week so as to best control for variations related to circadian rhythms or other activities of the participants.

All measurements were performed in a quiet room with dimmed lighting conditions. After a 20-min adaptation period, standard HRV resting recordings were collected for 5-min at rest before of the stimulation. Participants were asked to sit with knees at a 90° angle, both feet flat on the floor, hands on thighs, with palms facing upward, and keep the eyes closed. After a brief interview, sensors and stimulation devices were attached to the subject’s body, and then the experimental session started. In the within-subject experiment, each session consisted of a preliminary HRV recorded in the resting phase (baseline, 5 min) and consecutive phases of an active/sham stimulation (S1, 10 min), separated by a 1-week interval.

These sessions differed only for the stimulation (active-taVNS *vs*. sham-taVNS) in two 1-week-apart sessions that were randomly assigned in a counterbalanced order across participants.

After the HRV-resting, the current intensity was determined by each participant by using the threshold method to adjust the intensity of taVNS/sham stimulation intensity according to the participant’s sensitivity.

This procedure systematically identifies the maximal comfortable stimulation levels for each individual, as in the studies by Yakunina, Kim & Nam (2017b), and Ventura-Bort et al. (2018). To adjust before each session of stimulation for each participant intensity, the stimulation started with an intensity of 0.2 mA, and increasing in each trial by 0.1 mA, until the participant clearly felt a tingling but not painful perception to selectively stimulate afferent mechanoreceptive Aβ-fibers but not pain-related Aδ-fibers. This procedure was conducted twice for each stimulation location and the average of the intensities rated as not painful was used as the stimulation threshold.

taVNS electrodes were placed on the cymba conchae and on the helix for the active and sham condition, respectively. The same parameters, except for the intensity and site of stimulation on the ear, were used during active-taVNS and sham-taVNS, thus ensuring participants’ blinding to the type of stimulation. After the taVNS setup was completed, the neurostimulation was administered for 10 min while participants completed a demographic questionnaire. HRV measurement was performed to measure cardiac vagal activity during the overall active/sham stimulation condition. Self-reported rating scales were administered to assess tension pre and post each experimental condition (active/sham stimulation).

### Data analysis

Outliers (less than 1% of the data) were removed. According to previous studies (Forte et al., 2021; Forte et al., 2022a), HRV data were log-transformed for approximately following normal distribution.

A repeated measures (rest, active and sham stimulation condition) ANOVA was conducted on all HRV indices as dependent variables. We set *p* = 0.05 as a statistical significance level. Age and gender, considered as moderators, were also tested.

## Results

Participants were stimulated with an average intensity of 1.2 mA (SD ± 0.4). For each participant, the intensity of stimulation levels was comparable for active and sham treatment (F_1,27_ = 2.90; *p* = 0.11). There was no significant difference in stimulation levels between males and females (F_1,27_ = 1.26; *p* = 0.28). In addition, no significant differences were found between males and females in baseline HRV measurements (F_1,27_ = 0.02; *p* = 0.88).

### HRV analysis

Table 1 summarizes results of analyses with log-transformed data on HRV for the three conditions (resting, active-taVNS and sham-taVNS).

**Table 1:** Mean and standard deviation of HRV parameters in the different experimental conditions.

HRV parameters	Resting	Active-taVNS	Sham-taVNS	F	*p*
RMSSD	3.38 (0.49)	3.57 (0.56)^a^^b^	3.38 (0.51)	5.65	0.006
SDNN	3.66 (0.36)	3.80 (0.35)^a^^b^	3.67 (0.28)	5.25	0.008
LF	6.71 (0.64)^a^^c^	7.00 (0.55)^a^	6.99 (0.53)^c^	4.47	0.02
HF	5.94 (1.01)^a^	6.28 (0.93)^a^^b^	5.83 (0.89)^b^	5.52	0.007

**Notes:**

a Resting *vs*. taVNS: *p* < 0.05.

b Sham *vs*. taVNS: *p* < 0.05.

c Sham *vs*. Resting: *p* < 0.05.

taVNS, transcutaneous auricular vagus nerve stimulation; SDNN, standard deviation of the RR intervals; RMSSD, root mean squared differences of successive RR intervals; LF, low frequency; HF, high frequencies.

Across the entire group, regardless of the stimulation pattern, heart rate indices significantly increase HRV compared to baseline (See Table 1). Moreover, significant differences were found between active-taVNS and sham-taVNS in several HRV indices. In the *time domain*, both RMSSD (F_2,54_ = 5.65; *p* = 0.006; η_p_^2^ = 0.17) and SDNN (F_2,54_ = 5.25; *p* = 0.008; η_p_^2^ = 0.16) indices significantly differ between the conditions. Higher RMSSD and SDNN values were reported in active-taVNS compared to both resting (RMSSDmean difference: 0.19, *p* = 0.01; SDNNmean difference: 0.14; *p* = 0.01) and sham-taVNS (RMSSDmean difference: 0.19; *p* = 0.01; SDNNmean difference: 0.14; *p* = 0.01). No differences were found between resting condition and sham-taVNS (*p* > 0.05).

In the *frequency domain*, considering HF (F_2,54_ = 5.52; *p* = 0.007; η_p_^2^ = 0.17), a higher index was reported in active-taVNS compared to both resting (mean difference: 0.33; *p* = 0.04) and sham-taVNS (mean difference: 0.44; *p* = 0.007) conditions. No differences were found between resting condition and sham-taVNS (*p* > 0.05).

Considering LF, significant differences were found (F_2,54_ = 4.47; *p* = 0.02; η_p_^2^ = 0.14) with higher LF value in both active-taVNS (mean difference: 0.27; *p* = 0.03) and sham-taVNS (mean difference: 0.28; *p* = 0.03) than resting condition. No significant differences were reported between active-taVNS and sham-taVNS (*p* = 0.95). Together, these results indicate that active-taVNS differently from sham-taVNS affected cardiac vagal activity in both sympathetic and parasympathetic heart rate components.

Considering LF/HF ratio, significant differences were found (F_2,54_ = 5.42; *p* = 0.007; η_p_^2^ = 0.16) with a higher value in sham-taVNS compared to both resting (mean difference: 0.06; *p* = 0.01) and active taVNS (mean difference: 0.08; *p* = 0.01) conditions.

### Self-reported side effect

Regarding side effects, the differences between the active-taVNS and sham-taVNS were not significant (F_1,27_ = 0.32; *p* = 0.57), allowing us to rule out possible confounding effects due to participants’ disposition under the two stimulation conditions. None of the volunteers reported any discomfort during and after the stimulation and no adverse effects occurred during the stimulation period, or within a week after each session. Overall, stimulation was well tolerated, participants reported a low level of tension (mean score of 27.91) and there were no significant differences between conditions (active/sham stimulation) (F = 0.28; *p* = 0.58).

## Discussion

This study demonstrated that, in contrast to sham-taVNS, active-taVNS might modulate the HRV of young healthy individuals, resulting in significantly better RMSSD, SDNN, and HF power values.

Studies have investigated the effects of vagus nerve stimulation on HRV, with controversial (Wolf et al., 2021) or null findings (Vosseler et al., 2020). For example, De Couck et al. (2017) found that taVNS significantly increased SDNN compared to baseline without the effects on RMSSD, HF, or LF/HF, while other have found an increase in these parameters reflecting cardiac vagal modulation (Bretherton et al., 2019; Gauthey et al., 2020; Geng et al., 2022; Keute et al., 2019). In contrast, some studies have indicated that taVNS significantly decreased the LF/HF ratio without significant effects on other indexes of HRV (Clancy et al., 2014; Weise et al., 2015). This discrepancy, as mentioned in the introduction, may be explained by the variability in study design, which might affect how results are interpreted (Badran et al., 2018b). Indeed, some studies compared active stimulation to a “stimulation off” sham condition (Clancy et al., 2014; De Couck et al., 2017). Pain sensation or stressful emotions caused by taVNS, as well as respiration, can affect HRV (Laborde, Mosley & Thayer, 2017) and thus might result in confounding variables that potentially affect the interpretation of results. Therefore, we stimulated the helix (*i.e*., relatively free of vagal afferents) with the same parameters for the sham-taVNS and active-taVNS conditions. In addition, in the present study, self-reported side effects showed no significant difference; therefore, it is assumed that the potential influence of irrelevant variables was controlled, with consequently increased comparability between the active and control stimulation. Accordingly, these aspects might explain the inconsistencies with previous studies.

Another difference in our findings compared with those of previous stimulation studies is ascribed to the temporal dynamics of taVNS modulation. We chose to stimulate for only 10 min, according to studies that found HR changes soon after taVNS onset (Keute et al., 2019; Sclocco et al., 2019; Sharon, Fahoum & Nir, 2021). Therefore, recordings of less than 10 min provide reliable HRV indices (Forte, Favieri & Casagrande, 2019; Forte et al., 2021; Forte et al., 2022a; Forte et al., 2022b; Melo et al., 2018; Munoz et al., 2015; Nussinovitch et al., 2012; Thong et al., 2003; Voss et al., 2009).

With 10 min of stimulation, in terms of indices for HRV, we observed a significant increase in RMSSD and SDNN for active-taVNS compared to sham-taVNS in the temporal domain. While SDNN reflects both sympathetic and parasympathetic influences, RMSSD is thought to represent vagally mediated HRV, and both are less influenced by changes in respiratory parameters than frequency indices.

Additionally, a significant change in the parasympathetic activity of HF was observed in the frequency domain when compared to sham-taVNS. Thus, we confirmed enhancement of the measurements of RMSSD, SDRR, and HF power described in healthy young people in a previous study (Geng et al., 2022) for short (5 min) taVNS stimulation.

Individual stimulation patterns, however, showed no differences between active-taVNS and sham-taVNS for LF and distinct LF/HF tendencies. On different autonomic pathways, short taVNS may have a specific impact. HF power is mediated largely *via* respiration and mainly reflects cardiac parasympathetic nerve activity, while LF is a more complex power hypothesized to indicate a measure of mainly cardiac autonomic outflow by baroreflexes, sympathetic drive, and other yet unidentified factors (Billman, 2013; Goldstein et al., 2011). We observed a tendency to see a higher absolute increase in LF power compared to the increase in HF power, which led to an increase in the LF/HF ratio, presumably due to already low sympathetic and high parasympathetic prevalence. The change in LF power or LF/HF ratio may occur not by affecting cardiac autonomic outflows directly but by affecting modulation of these outflows by baroreflexes, as has been shown (Antonino et al., 2017).

Moreover, the difference between sham-taVNS and baseline, in both LF and LF/HF, could suggest higher sympathetic activity during sham stimulation. Also, active-taVNS improves sympathetic activity compared to baseline but, in concomitance, it involves an increase in parasympathetic activity. This suggests an activating role of stimulation, which should be controlled. Additionally, Geng et al. (2022) proposed that the baseline LF/HF ratio was a significant predictor of participants’ responses to taVNS: a higher baseline LF/HF ratio was associated with a greater LF/HF ratio decrease. This evidence could be better analyzed in further studies despite the issues related to these measures. Indeed, the physiological source and meaning of LF power are difficult to discern, justifying agreement among the scientific community that HF is the most effective and reliable index of the frequency domain in the interpretation of HRV (Billman, 2013; von Rosenberg et al., 2017). Such predictions could enable the selection of optimal individuals for taVNS, considering the number of conditions that influence sympathetic prevalence/autonomic imbalance, *e.g*., pain, inflammation, and even position.

Studies on the effect of taVNS on LF and LF/HF have provided controversial results (Wolf et al., 2021). Interestingly, De Couck et al. (2017) found no effects on LF/HF for short (10 min) stimulation, but taVNS significantly increased LF and LF/HF in prolonged stimulation (35 min), suggesting a period of ‘adaptation’ to stimulation. An increased LF/HF ratio was confirmed after longer stimulation (60 min) (Tran et al., 2019) but not after brief stimulation (5 min) (Geng et al., 2022). By contrast, other studies have indicated that taVNS significantly decreases the LF/HF ratio without significant effects on other indexes of HRV (Clancy et al., 2014; Weise et al., 2015). Notably, these studies compared different stimulation targets and parameters, which might affect interpretation of the results.

Recently, the effect of specific taVNS parameters on these markers of parasympathetic vagal activity has been examined. Lower pulse duration values (<500 μs) seem to allow for a more selective nerve fiber type of recruitment (Machetanz et al., 2021). These results support the role of the choice of stimulation parameters, considering that the vagus nerve comprises myelinated and unmyelinated fibers with disparate diameters and activation thresholds (Deuchars et al., 2018). In a study by Machetanz et al. (2021), variations in pulse width parameters corresponded to changes in stimulation intensity. In contrast, we adjusted the latter according to the participants’ perceptual threshold.

In conclusion, our study can be considered as part of a dynamic and developing empirical background. Although several authors have explored the effects of taVNS and speculated on its neurophysiological mechanism, the direction of the results is unclear.

This study provides evidence that taVNS may increase cardiac vagal activity, although the findings should be considered with caution. taVNS effects are indirect considering that the auricular branch of the vagus nerve consists only of afferent fibers. Specifically, as proposed by some authors (Komisaruk & Frangos, 2022; Murray et al., 2016; Sawchenko, 1983), taVNS may increase input to the NTS, thereby increasing the activity of NTS neurons projecting to the two vagal efferent nuclei: the dorsal motor nucleus and the nucleus ambiguus. Increased activation in these nuclei may, in turn, increase vagal control of cardiac activity (Komisaruk & Frangos, 2022; Sawchenko, 1983).

taVNS may have neuromodulatory effects on vmHRV that are not mediated by the NTS but instead *via* sensory afferent projections to the upper cervical spinal cord (Mahadi et al., 2019). Similarly, increased vagal control of HRV during auricular stimulation correlates with frequency-specific increases and decreases in oscillatory activity in various brain areas (*i.e*., frontal and frontoparietal areas (Machetanz et al., 2021)). Finally, taVNS appears to increase spontaneous cardiac baroreflex sensitivity and indirectly influence parasympathetic efferent innervation of the heart (Antonino et al., 2017).

However, despite the anatomical and physiological plausibility of the indirect effects of taVNS on HRV, many steps to validation remain before HRV may be considered a relevant index of taVNS efficacy. Considering the evidence provided for both mental health and cognitive functions of taVNS (Colzato, Ritter & Steenbergen, 2018b; Thakkar et al., 2020) and HRV (Forte, Favieri & Casagrande, 2019; Forte et al., 2021; Forte et al., 2022a; Forte et al., 2022b), these results open the way to potential clinical trials.

### Limitations

Despite the encouraging results, this study has limitations. The complex nature of LF power could explain the results (Shaffer, McCraty & Zerr, 2014). LF oscillations provide information about blood pressure control mechanisms, such as the modulation of vasomotor tone (Berntson et al., 1997; Taylor & Sarno, 1998). HF and LF power showed rather slight changes of conditions compared to baseline, which suggests that markers of overall HRV are more sensitive to a presumable autonomic activation than specific increases following taVNS, presumably due to already low sympathetic and high parasympathetic prevalence. Therefore, unclear knowledge about the dominance of HRV indices, in particular LF, has been highlighted, as they may be inaccurate measures of SNS activity and of SNA in general (Laborde, Mosley & Thayer, 2017).

Despite the lack of a clear consensus, to optimize the effects of taVNS-related HRV, we stimulated the cymba conchae of the left ear. However, some taVNS studies have shown significant results for right tragus stimulation (Badran et al., 2018b; Badran et al., 2022; Yakunina, Kim & Nam, 2017b). Considering safe parameters, conventionally, the left side is the preferred stimulation site due to concerns about cardiac side effects (Borges, Laborde & Raab, 2019; Burger et al., 2019). However, it might be interesting to test the short-term effects of adopting variations of stimulation target (left/right, cymba/tragus).

In accordance with the range suggested by recent recommendations for experiment planning with HRV in psychophysiological research, it is unnecessary to use recordings longer than 120 s to obtain accurate measures of RMSSD (Laborde, Mosley & Thayer, 2017). However, future studies might test the long-term effects of taVNS on HRV parameters, *e.g*., in multiple stimulation sessions spread over a longer period (longer than a 24-h period). Furthermore, the mediating role of physiological covariates, such as baroreflex and respiratory changes as well as blood pressure, could be evaluated in further studies. Moreover, vmHRV could be compared with additional appropriate biomarkers of taVNS efficacy, such as somatosensory evoked potentials (Fallgatter et al., 2003), pupillary dilation, event-related potential P300, and salivary alpha-amylase (Burger et al., 2019; Warren et al., 2019).

Finally, although the study employed a within-subject crossover design, we adopted a straight control of taVNS intensity for each participant and measured the absence of gender differences in resting HRV parameters, the study is not exempt from gender limitations due to recruitment from a female-dominated field. Future studies may answer the question of gender differences with a more heterogeneous sample.

## Conclusion

In conclusion, our results indicate a rapid increase in several HRV parameters when taVNS is used to recruit fibers in the cymba compared with taVNS stimulation administered *via* the helix. Future studies should examine the effect with a heterogeneous sample and with other appropriate biomarkers to support our findings. A shortened stimulation time may be useful to evaluate the acute effects of taVNS on HRV, which may be used as a physiological indicator of autonomic tone for safe clinical applicability. Investigation of taVNS-mediated changes in brain networks that promote cardiac activity is necessary to better understand the physiological mechanism of action of taVNS and to establish meaningful protocols for research and clinical trials.

## Supplemental Information

10.7717/peerj.14447/supp-1 Supplemental Information 1 Data.

## Additional Information and Declarations

### Data Availability

The following information was supplied regarding data availability: The raw data is available in the Supplemental File.
